# The effect of constitutive representations and structural constituents of ligaments on knee joint mechanics

**DOI:** 10.1038/s41598-018-20739-w

**Published:** 2018-02-02

**Authors:** Gustavo A. Orozco, Petri Tanska, Mika E. Mononen, Kimmo S. Halonen, Rami K. Korhonen

**Affiliations:** 1 Department of Applied Physics, University of Eastern Finland, Kuopio, Finland; 2 Department of Health Science and Technology, Aalborg University, Aalborg, Denmark

## Abstract

Ligaments provide stability to the human knee joint and play an essential role in restraining motion during daily activities. Compression-tension nonlinearity is a well-known characteristic of ligaments. Moreover, simpler material representations without this feature might give reasonable results because ligaments are primarily in tension during loading. However, the biomechanical role of different constitutive representations and their fibril-reinforced poroelastic properties is unknown. A numerical knee model which considers geometric and material nonlinearities of meniscus and cartilages was applied. Five different constitutive models for the ligaments (spring, elastic, hyperelastic, porohyperelastic, and fibril-reinforced porohyperelastic (FRPHE)) were implemented. Knee joint forces for the models with elastic, hyperelastic and porohyperelastic properties showed similar behavior throughout the stance, while the model with FRPHE properties exhibited lower joint forces during the last 50% of the stance phase. The model with ligaments as springs produced the lowest joint forces at this same stance phase. The results also showed that the fibril network contributed substantially to the knee joint forces, while the nonfibrillar matrix and fluid had small effects. Our results indicate that simpler material models of ligaments with similar properties in compression and tension can be used when the loading is directed primarily along the ligament axis in tension.

## Introduction

Stability of the knee joint is provided by different structures such as ligaments, menisci, and muscles which exhibit a complex mechanical behavior and affect the articular cartilage response under different loading conditions^1^. In particular, ligaments play an essential role in providing stability in more than one degree of freedom as well as restraining knee joint motion during external loads. However, the contributions of the individual ligaments and their structural constituents, and the importance of the material models of ligaments are not well known^2–4^. Research on the effect of these ligament properties in the knee joint contributes to understanding joint disorders and injury mechanisms.

The experimental and clinical studies have been complemented with computational knee models to overcome inherent limitations such as high cost, difficulties to obtain accurate measures *in vivo*, and reproduce degenerative situations in the knee. Previous numerical knee models have investigated the mechanical behavior of knee joint ligaments under different loading conditions^5–16^. For instance, some studies simulated ligaments as nonlinear elastic springs and cartilage and menisci as a simple linear elastic material or rigid^12,17–19^. Other studies have included realistic geometries of ligaments, but cartilage and menisci were treated as linear elastic materials^14,20^. Models with more complicated properties for cartilage have not typically incorporated fibril-reinforced biphasic properties for ligaments during dynamic gait^21,22^.

Nonlinear tensile properties of ligaments along axis have been extensively documented^23–25^, however, less studies have reported the anisotropy and the compressive mechanical response of the tendon or ligament tissue^26–28^. Nonetheless, the anisotropy of these tissues has been primarily suggested to results from the collagen fibril orientation^26^. During *in vivo* loading, the contribution of the compressive properties of ligaments may occur to some extent when ligaments are exposed to multiaxial states of stresses, bending, and transverse compressive loads. However, many finite element (FE) studies^4,12,14,29,30^ and musculoskeletal models^18,31^ have considered that, due to the ligament and tendon structure and composition, their contribution in tension is much greater than that in compression. This variance in compression and tension along the main axis of loading (strong in tension and soft in compression) has also been documented thoroughly for other fibril-reinforced poroelastic tissues, such as articular cartilage and meniscus^32–36^.

In order to apply knee joint models for clinical cases with large patient groups, they should be simple and fast but at the same time accurate and reliable. Because ligaments are primarily in tension during joint loading, it might be that the aforementioned compression-tension characteristic is not always needed. Then the properties along the tensile direction mainly control the ligament response and the compressive properties are not necessarily that important. For this reason, ligaments modeled in the knee with similar properties in compression and tension might give reasonable results at those time points of loading when there is minimal amount of bending and therefore local compression. This approach would simplify the model generation applied for clinical purposes, reducing computational demands (run time) and labour required to generate personalized knee models. Specifically, reliable and simplified models could potentially provide expeditious diagnostics for improving clinical outcomes in patients with orthopedic disorders.

Thus, the aim for this study was to investigate the effect of different constitutive representations and structural constituents (fibrillar matrix, nonfibrillar matrix, fluid) of ligaments on knee joint mechanics during the stance phase of gait. A finite element model of the knee joint which takes into account geometric and material nonlinearities of meniscus and cartilages was applied. Particularly, we simulated the effect of fibril-reinforced porohyperelastic properties of ligaments on the knee joint function as well as forces, stresses and strains in the tibial and patellar cartilages during walking. These results were compared with knee models with simpler geometries (springs) and 3-D constitutive models for ligaments. We hypothesize that the collagen network of ligaments contributes strongly to the knee joint mechanics while the contribution of the nonfibrillar matrix and especially fluid is minimal. However, we also hypothesize that at certain time points of stance when ligaments are elongated primarily along their axis it is possible to obtain similar mechanical response with a simple representation for ligaments compared to a complex formulation (fibril-reinforced porohyperelastic).

## Methods

**Figure 1:**
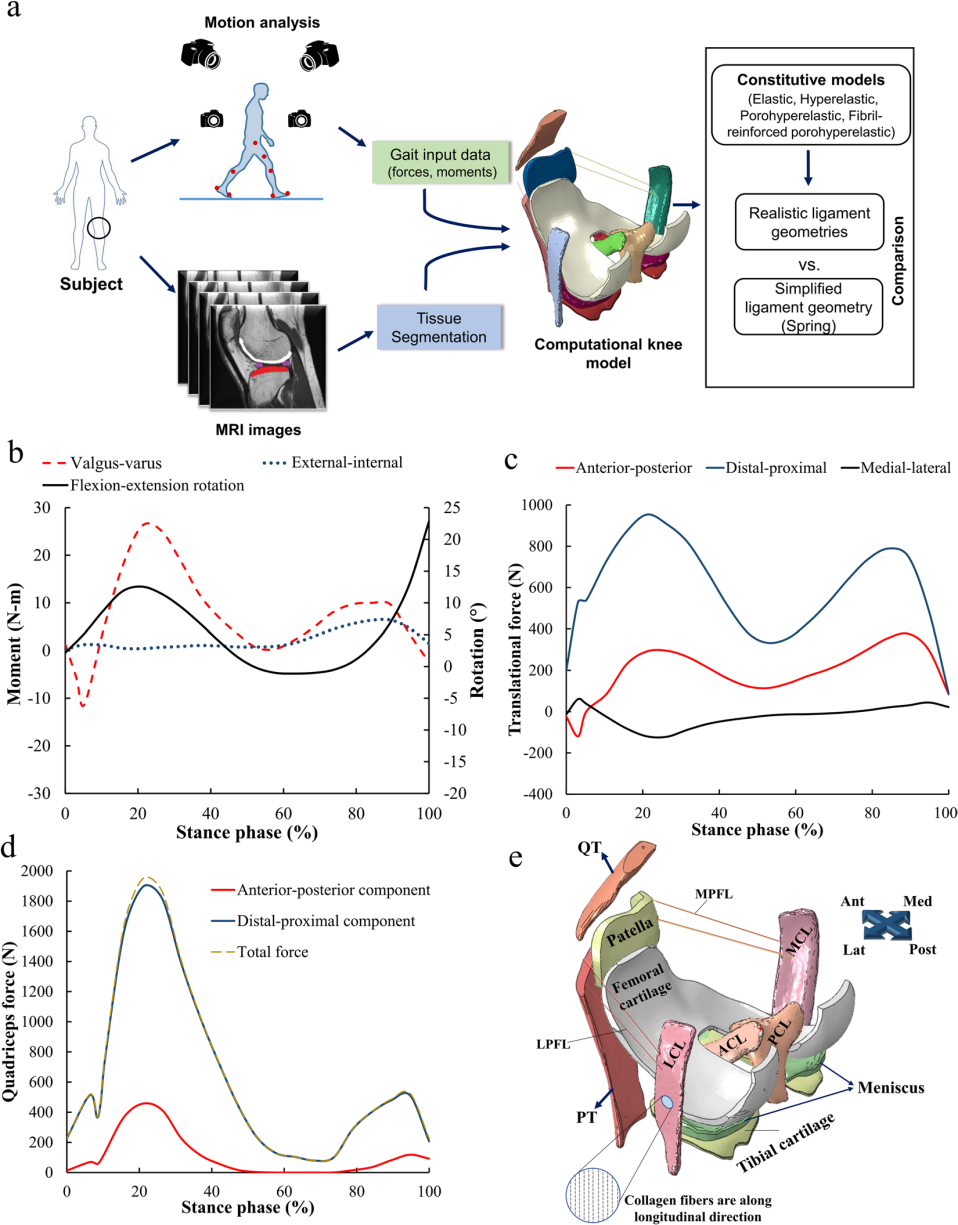
(**a**) Study workflow. (**b**) Gait input data for the numerical knee joint model, as obtained directly from^21^. (**c**) External-internal, valgus-varus moments, and flexion-extension rotation. (**d**) Anterior-posterior, distal-proximal and medial-lateral forces. Components of the total quadriceps force. (**e**) A posterior-lateral view of the three-dimensional finite element model of the knee shows articular cartilages, ligaments and tendons. The original model was compared to experiments in^21^.

A general workflow of this study is shown in Fig. 1a.

### Finite element model

A previously developed finite element model was applied and modified here^22^. A knee joint of an asymptomatic male subject (28 years, 80 kg) was imaged using MRI and the joint tissues (femoral, tibial and patellar cartilages, and meniscus) were segmented and meshed as described in our earlier study^22^. This MR imaging was conducted according to the ethical guidelines of Kuopio University Hospital, Finland. MR imaging was conducted with the permission (94/2011) from the local ethical committee of the Kuopio University Hospital, Kuopio, Finland, and written informed consent was obtained from the volunteer. Based on the same knee joint, patellar and quadriceps tendons (PT and QT) and six ligaments were segmented in this study. The six ligaments were anterior and posterior cruciate ligaments (ACL and PCL), medial and lateral collateral ligaments (MCL and LCL), and medial and lateral patellofemoral ligaments (MPFL and LPFL) (Fig. 1). Ligament and tendon geometries were segmented using the MR images (a clinical 3.0 T scanner, Philips, Best, Netherlands) which were taken using 3D fast spin-echo sequence (VISTA) (in-plane resolution = 0.5 mm, slice thickness = 0.5 mm, TR = 1300 ms, TE = 32.3 ms). Ligaments and their exact insertion sites were determined by consulting two orthopedic surgeons during the segmentation process. The segmented geometries were meshed in Abaqus v.6.13 (Dassault Systémes, Providence, USA). Gait input, properties of the patellofemoral ligaments and boundary conditions for the knee model were identical to an earlier study^21,22^ (Fig. 1b–d). In the simplified models, the ligaments and tendons were represented using an array of spring elements^22,37^ or meshed with tetrahedral pore pressure elements (type = C3D4P) in the 3D continuum models. In the most complex model, ACL, PCL, LCL and MCL were modeled as a fibril-reinforced porohyperelastic material. Cartilages were defined as a fibril-reinforced poroviscoelastic material^38–40^, while menisci were considered as a fibril-reinforced porohyperelastic material; our previous studies have validated the materials and applied these to the knee joint models^37,41^. The cartilage-bone interfaces were defined as rigid boundary conditions. Frictionless surface-to-surface contact was defined for all the contacting surfaces, i.e., interactions between cartilages and menisci, the external surfaces of solid ligaments and cartilages, and ACL and PCL. The master surfaces were determined as a surface, whereas the slave surfaces were defined as a node surface. Numerical simulations were run on a high performance workstation with 48x Intel Xeon E5-2690 v3 CPU (2.60 GHz) and 264 GB of memory.

### Models with different mechanical properties for ligaments

Five knee joint models were constructed with different constitutive models for the ligaments: 1) spring, 2) linear elastic, 3) hyperelastic, 4) porohyperelastic and 5) fibril-reinforced porohyperelastic (FRPHE) material, which we briefly describe here (see the supplementary material for more details about the implementation of these constitutive models). In all cases, stress-strain behavior of the ligaments was defined such that the ligaments produced force in tension (strain > 0) but not in compression (strain < 0) during the gait. For the first two cases, Hooke´s law represents the relation between stresses (or forces) and strains (or elongations)

1 $${{\boldsymbol{\sigma }}}_{{\rm{tot}}}={\bf{C}}\,{\boldsymbol{\varepsilon }},$$

where ${{\boldsymbol{\sigma }}}_{{\bf{t}}{\bf{o}}{\bf{t}}}$ is the Cauchy stress tensor, **ε** is the infinitesimal strain tensor, and **C** is the fourth-order stiffness matrix, which is defined by the Young’s modulus (*E*) and Poisson’s ratio (*ν*). For the spring model, the stiffness is defined by a spring constant *k*_s_. The third model was defined using a neo-Hookean material, in which the stresses are given by

2 $${{\boldsymbol{\sigma }}}_{{\rm{tot}}}={K}_{{\rm{m}}}\frac{\mathrm{ln}(J)}{J}{\bf{I}}+\frac{{G}_{{\rm{m}}}}{J}({\bf{F}}\cdot {{\bf{F}}}^{{\rm{T}}}-{J}^{\frac{2}{3}}\,{\bf{I}}),\,$$

where *K*_m_ is the bulk modulus, *G*_m_ is the shear modulus, *J* is the determinant of the deformation gradient tensor **F** and **I** is the unit tensor. For the fourth model, the ligaments were described as a biphasic tissue in which the porous solid matrix is fully saturated with water. The total stress in the tissue is then given by

3 $${{\boldsymbol{\sigma }}}_{{\rm{tot}}}={{\boldsymbol{\sigma }}}_{{\rm{s}}}+{{\boldsymbol{\sigma }}}_{{\rm{fl}}}={{\boldsymbol{\sigma }}}_{{\rm{eff}}}-p{\bf{I}},$$

where ${{\boldsymbol{\sigma }}}_{{\rm{tot}}}$ is the total stress tensor, ${{\boldsymbol{\sigma }}}_{{\rm{s}}}$ is the stress in the solid matrix, ${{\boldsymbol{\sigma }}}_{{\rm{fl}}}$ is the stress in the fluid matrix, *p* is the hydrostatic pressure and ${{\boldsymbol{\sigma }}}_{{\rm{eff}}}$ is the effective solid stress. In this model, ${{\boldsymbol{\sigma }}}_{{\rm{eff}}}$ was described by equation (2). Additionally, the permeability $k$ was assumed to be strain-dependent and is as follows:

4 $$k={k}_{0}{[\frac{{\varphi }_{0}{\varphi }_{{\rm{f}}}}{(1-{\varphi }_{0}){\varphi }_{{\rm{s}}}}]}^{2}\exp (\frac{M({J}^{2}-1)}{2}),\,$$

where *k*_0_ is the initial permeability, *M* is a positive constant, *ϕ*_0_ is the initial volume fraction of the solid phase, *ϕ*_f_ is the current volume fraction of the fluid phase and *ϕ*_s_ is the current volume fraction of the solid phase.

Finally, the FRPHE model considers that the solid matrix is divided into a non-fibrillar part, describing primarily the proteoglycan matrix, and a fibrillar elastic network, representing the collagen fibers. The total stress in the ligament tissue is then given by

5 $${{\boldsymbol{\sigma }}}_{{\bf{t}}{\rm{ot}}}={{\boldsymbol{\sigma }}}_{{\rm{s}}}-p{\bf{I}}={{\boldsymbol{\sigma }}}_{{\rm{f}}}+{{\boldsymbol{\sigma }}}_{{\rm{nf}}}-p{\bf{I}},$$

where ${{\boldsymbol{\sigma }}}_{{\rm{f}}}$ and ${{\boldsymbol{\sigma }}}_{{\rm{nf}}}$ are the stresses in the collagen fibers and the non fibrillar matrix, respectively. The non-fibrillar component of the ligament is defined using a neo-Hookean material with biphasic properties as was described in equation (3). The fibril stress ${\sigma }_{{\rm{f}}}$ is given by

6 $${\sigma }_{{\rm{f}}}=\{\begin{array}{ll}{E}_{{\rm{f}}}{\varepsilon }_{{\rm{f}}}\,, & {\varepsilon }_{{\rm{f}}} > 0\\ 0\,, & {\varepsilon }_{{\rm{f}}}\le 0\end{array},$$

where *E*_f_ is the fibril network modulus and *ε*_f_ is the fibril strain. The fibril network stress arises from the sum of primary and secondary collagen fibril stresses, which is calculated separately for each integration point in each element^40^. Stresses for these fibrils in tension were

7 $$\{\begin{array}{c}{\sigma }_{{\rm{f}},{\rm{p}}}={\rho }_{z}C{\sigma }_{{\rm{f}}}\\ {\sigma }_{{\rm{f}},{\rm{s}}}={\rho }_{z}{\sigma }_{{\rm{f}}}\end{array},$$

where ${\sigma }_{{\rm{f}},{\rm{p}}}$ and ${\sigma }_{{\rm{f}},{\rm{s}}}$ are the fibril stresses for primary and secondary fibrils, respectively, *C* is the density ratio between primary and secondary fibrils and ${\rho }_{z}$ is the relative collagen density.

### Analysis

**Figure 2:**
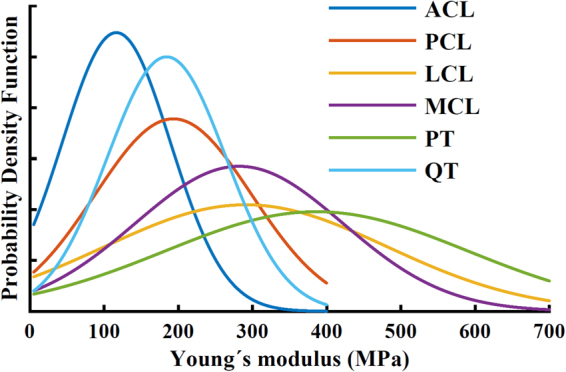
Probabilistic density function of Young’s modulus of the **ACL**^2,15,25,75–83^, **PCL**^2,15,75,77,80,82,84–87^, **MCL**^2,16,24,25,42,88–94^, **LCL**^2,23,88,91,92,94–101^, **PT**^81,88,95,102–114^, and **QT**^81,112,113,115^. In this study, biphasic properties for ligaments were selected based on^43^ and **MPFL** and **LPFL** were defined as elastic truss elements with a Young’s modulus equal to 19 MPa and Poisson’s ratio was set to 0.499.

For the initial analysis, we estimated the initial group of material constants for each constitutive model based on experimental studies (Table 1). A Gaussian distribution was generated with a relative standard deviation (RSD) of 0.62, 0.60, 0.70, 0.71, 0.52 and 0.43 for the ACL, PCL, LCL, MCL, PT and QT (Fig. 2) ^14,16,42,43^. Based on these constants we performed a preliminary evaluation for all models on the tibial reaction force. A detailed list of material parameters used in the initial analysis for each constitutive model is given in Table 1.

**Table 1:** Reference values for ligament material parameters. In the spring and FRPHE models, the adjusted values are the fibril network modulus values after matching the first peak of the tibiofemoral joint reaction force with the rest of the knee models.

Material formulation	ACL	PCL	MCL	LCL	PT	QT
**Spring**						
*Initial*						
*k* (Nmm^−1^)	201	258	114	134	545	475
*Adjusted*						
*k* (Nmm^−1^)	100	129	57	67	545	475
**Linear Elastic**						
*E* (MPa)	123	168	224	280	336	370
ν	0.4	0.4	0.4	0.4	0.4	0.4
**Hyperelastic** (**Neo-Hookean**)						
*C*_1_ (MPa)	22	30	40	50	60	66
*D* (MPa^−1^)	0.005	0.0036	0.003	0.0021	0.002	0.002
**Porohyperelastic**						
*C*_1_ (MPa)	22	30	40	50	60	66
*D* (MPa^−1^)	0.005	0.0036	0.003	0.0021	0.002	0.002
*k*_0_ (10^−15^ m^4^/Ns)	2.9	2.9	2.9	2.9	2.9	2.9
*M*	7.98	7.98	7.98	7.98	7.98	7.98
**Fibril-reinforced porohyperelastic** (**FRPHE**)						
*Initial*						
*E*_f_ (MPa)	130	180	270	280	184	380
*Adjusted*						
*E*_f_ (MPa)	100	40	120	100	150	150
*Unchanged values*						
*E*_m_ (MPa)	1	1	1	1	10	10
ν_m_	0.4	0.4	0.4	0.4	0.4	0.4
*k*_0_ (10^−15^ m^4^/Ns)	2.9	2.9	2.9	2.9	2.9	2.9
*M*	7.98	7.98	7.98	7.98	7.98	7.98
*C*	12	12	2	2	2	2

*k*_s_ = spring constant, *E* = Young’s modulus, ν = Poisson’s ratio, *C*_1_ = material coefficient, *D* = compressibility coefficient, *k*_0_ = initial permeability, *M* = exponential term for the strain-dependent permeability, *E*_m_ = nonfibrillar matrix modulus, *E*_f_ = fibril network modulus, ν_m_ = Poisson’s ratio of the nonfibrillar matrix, *C* = density ratio between primary and secondary fibrils.

After the preliminary assessment of the mechanical parameters for every constitutive model, we modified the stiffness in the spring model and the fibril network modulus in the FRPHE model iteratively in the ligaments until the obtained “adjusted” values matched the first peak of the tibial reaction force with respect to the rest of the knee models. Then, the FRPHE model was used for evaluating the influence of the fibril network modulus and the nonfibrillar matrix modulus of ligaments. Furthermore, the initial permeability *k*_0_ and material constant *M* were also varied in this analysis. Finally, in the biphasic models, we analyzed two boundary conditions on the external surface of ligaments: sealed and free draining. A list of the ranges of the material parameters used in the parametric study is given in Table 2.

**Table 2:** Range of values used in the parametric analysis with the FRPHE model.

Fibril network modulus, *E*_f_	10–250 (MPa)
Nonfibrillar matrix modulus, *E*_m_	1–50 (MPa)
Poisson’s ratio, *ν*_m_	0.4
Initial permeability, *k*_0_	0.15–15 (10^−15^ m^4^/Ns)
Nonlinear term for the strain-dependent permeability, *M*	1–10

In order to assess the effect of the ligament representation (springs and 3D continuum) and the contributions of different structural constituents (fibril network, nonfibrillar matrix, fluid), knee joint reaction forces, rotations and translations as well as stresses, strains and pore pressures of the tibiofemoral and patellofemoral contact were determined for all models during the stance phase of gait.

## Results

### Comparison between the constitutive models

**Figure 3:**
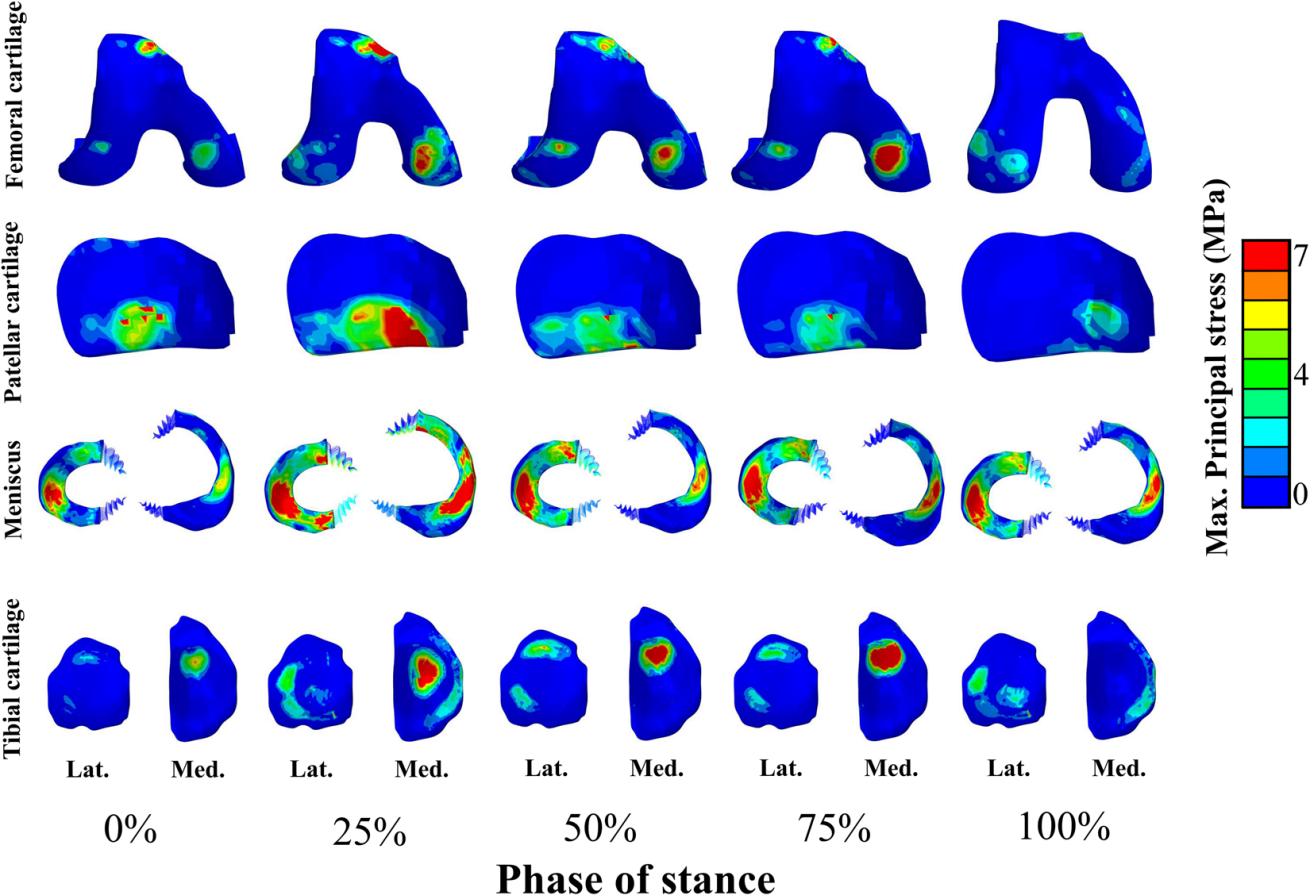
Maximum principal stress distributions of femoral, patellar, and tibial cartilages, and meniscus in the FRPHE model during the stance phase of gait (Lat: lateral; Med: medial).

**Figure 4:**
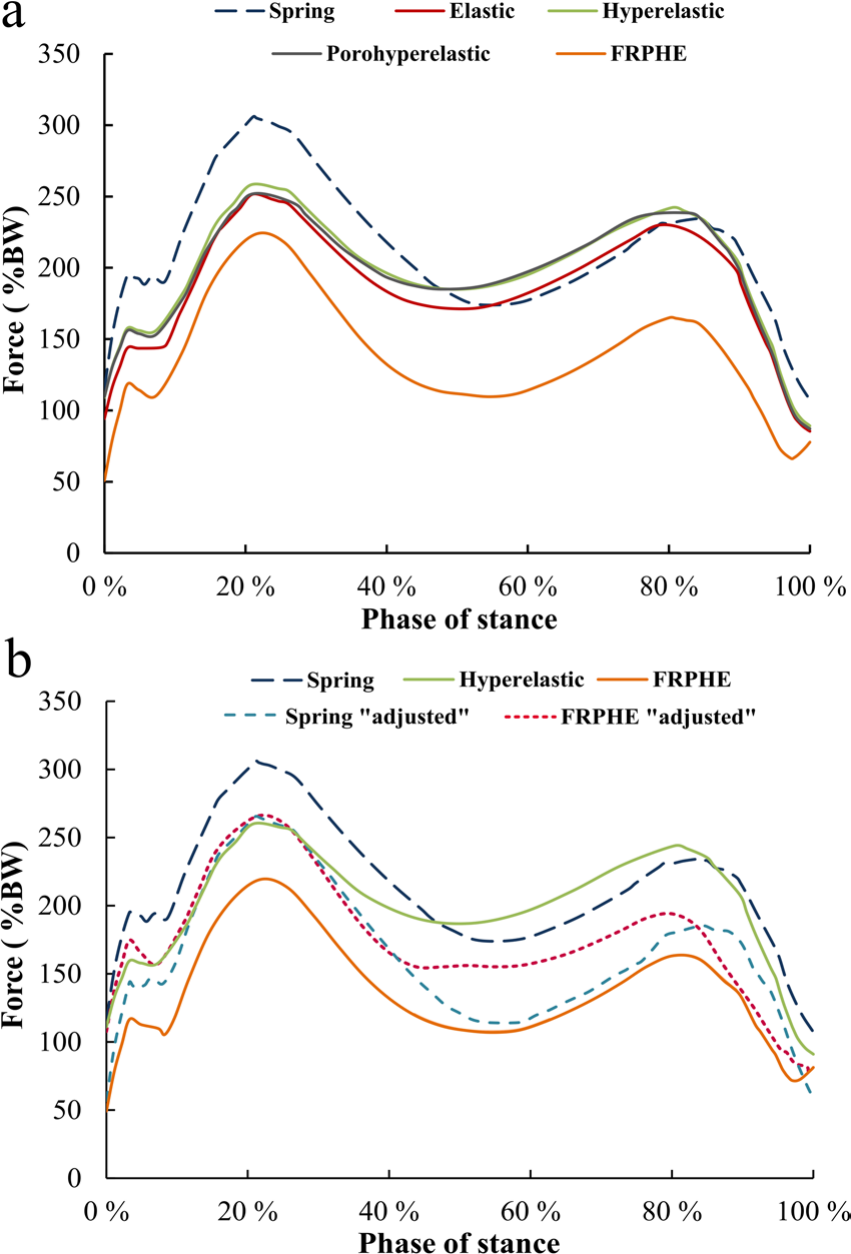
(**a**) The total tibiofemoral joint reaction force for the models with different ligament representations (Table 1). (**b**) The total joint forces with the “adjusted” material parameters for the FRPHE model and the spring model (Table 1).

**Figure 5:**
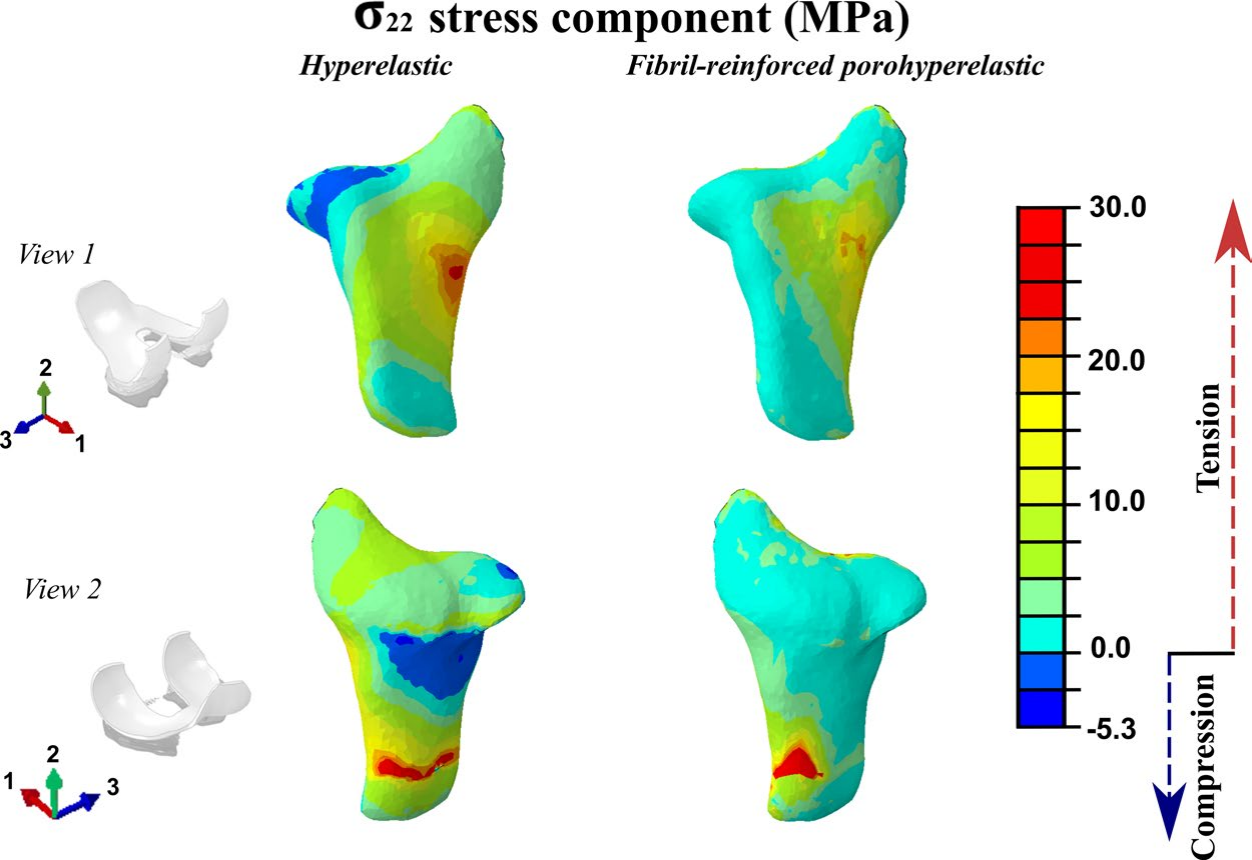
Tensile and compressive stresses for *σ*_22_ in PCL for the hyperelastic (left) and fibril-reinforced poroelastic material (right) at the second peak of the stance phase. The hyperelastic model experiences both tensile (positive values) and compressive (negative values) stresses, though the tensile stresses are dominant, while the FRPHE model exhibited tensile stresses and virtually no compressive stresses.

Stress distribution in cartilages and menisci at different phases of stance is seen in Fig. 3. By using the values of the material parameters in the preliminary analysis (Table 1), tibial reaction forces in the models with elastic, hyperelastic and porohyperelastic properties for ligaments showed similar behavior throughout the stance, while the model with ligaments modeled as spring elements and FRPHE properties yielded a similar trend, but the last case exhibited lower joint reaction forces in terms of body weight (BW) during the entire stance phase of gait (Fig. 4a). After this first evaluation, we matched the first peak force among the models through a decrease of 50% of the stiffness for all ligaments in the spring model as well as modifying the fibril network modulus values in the FRPHE model (Table 1). For these “adjusted” values, both the modified spring and FRPVE models yielded lower joint reaction forces after the first peak load (Fig. 4b), and the spring model exhibited slightly lower forces in the mid-stance of the gait compared to the FRPHE model. Additionally, the model with the FRPHE ligaments displayed high tensile stresses and essentially no compressive stresses, while the other models with solid ligaments experienced both tensile and compressive stresses at the second half of the stance phase (Fig. 5).

**Figure 6:**
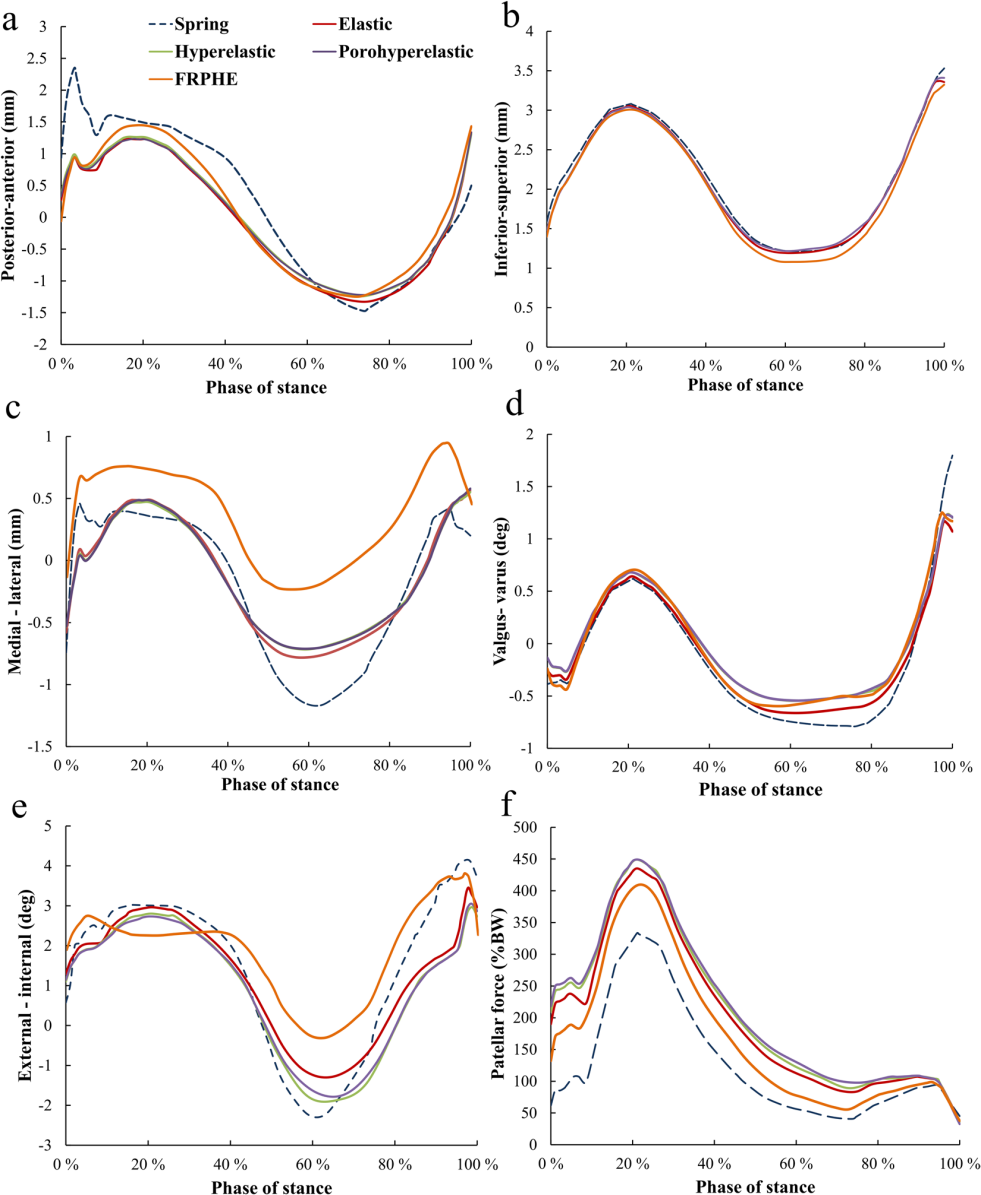
Tibial translations (**a**–**c**), rotations (**d**,**e**) with respect to femur and patellar forces (**f**) during the stance phase of gait for different constitutive models for the ligaments. (**a**) Posterior-anterior translation. (**b**) Inferior-superior translation. (**c**) Medial-lateral translation. (**d**) Valgus-varus rotation. (**e**) External-internal rotation. (**f**) Patellar force.

Based on these models with “adjusted” material parameters, inferior-superior translations and varus-valgus rotations in the knee were similar in all models. However, the models with solid ligaments yielded a maximum reduction of 73% (1.72 mm) in the anterior femoral translation, as compared to the spring model, at the beginning of stance (Fig. 6). The medial-lateral translation was similar in the solid models with exception of the FRPHE model for ligaments, where lateral translation was higher throughout the stance (Fig. 6c). Though, the change in the translation during the stance (min – max) was similar in all solid models. Compared to the solid models, the spring model showed slightly higher medial translation at 60% of stance. Further, the FRPHE model displayed a smaller external-internal rotation at ~60% of the stance. In the FRPHE model, the patellar force was ~24% higher at ~25% of the stance compared with the spring model (Fig. 6e and f). Again, all other solid models gave consistent results and these forces were slightly higher than those in the FRPHE and spring models.

**Figure 7:**
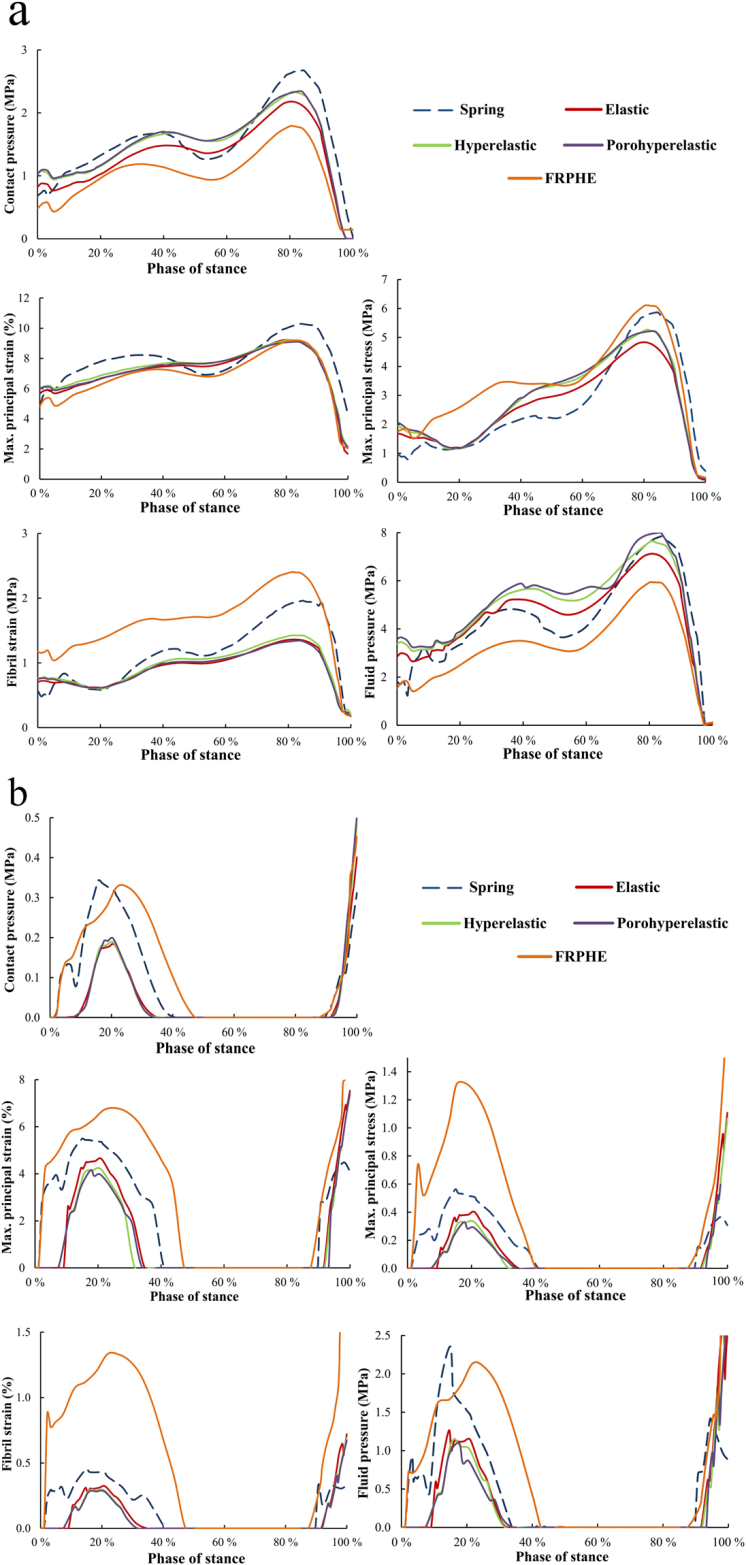
Average contact pressure, maximum principal strain, maximum principal stress, fibril strain, and fluid pressure in the contact area of the (**a**) medial and (**b**) lateral tibial cartilage surfaces during the stance phase of gait for different constitutive models of the ligaments.

Similarly with joint reaction forces and motions, elastic, hyperelastic, and porohyperelastic models displayed quite similar behavior in the quantitative analysis of average contact pressures, maximum principal strains and stresses, fibril strains and fluid pressures in the contact area of the medial and lateral compartments of tibial cartilage during the entire stance phase of gait (Fig. 7). In contrast, the FRPHE model revealed lower values of contact and fluid pressures, and higher values of maximum principal stresses and fibril strains in the medial compartment, while contact and fluid pressures, stresses and strains in the lateral compartment were mostly highest in the FRPHE model. This result was related to the different contact area in the FRPHE model resulting from slightly different medial-lateral translation and external-internal rotation (Fig. 6).

The run time for the spring model was 605 minutes, whereas those for the hyperelastic and FRPHE models were 331 and 823 minutes, respectively.

### Parametric analysis of the effect of fibrillar and non-fibrillar components

**Figure 8:**
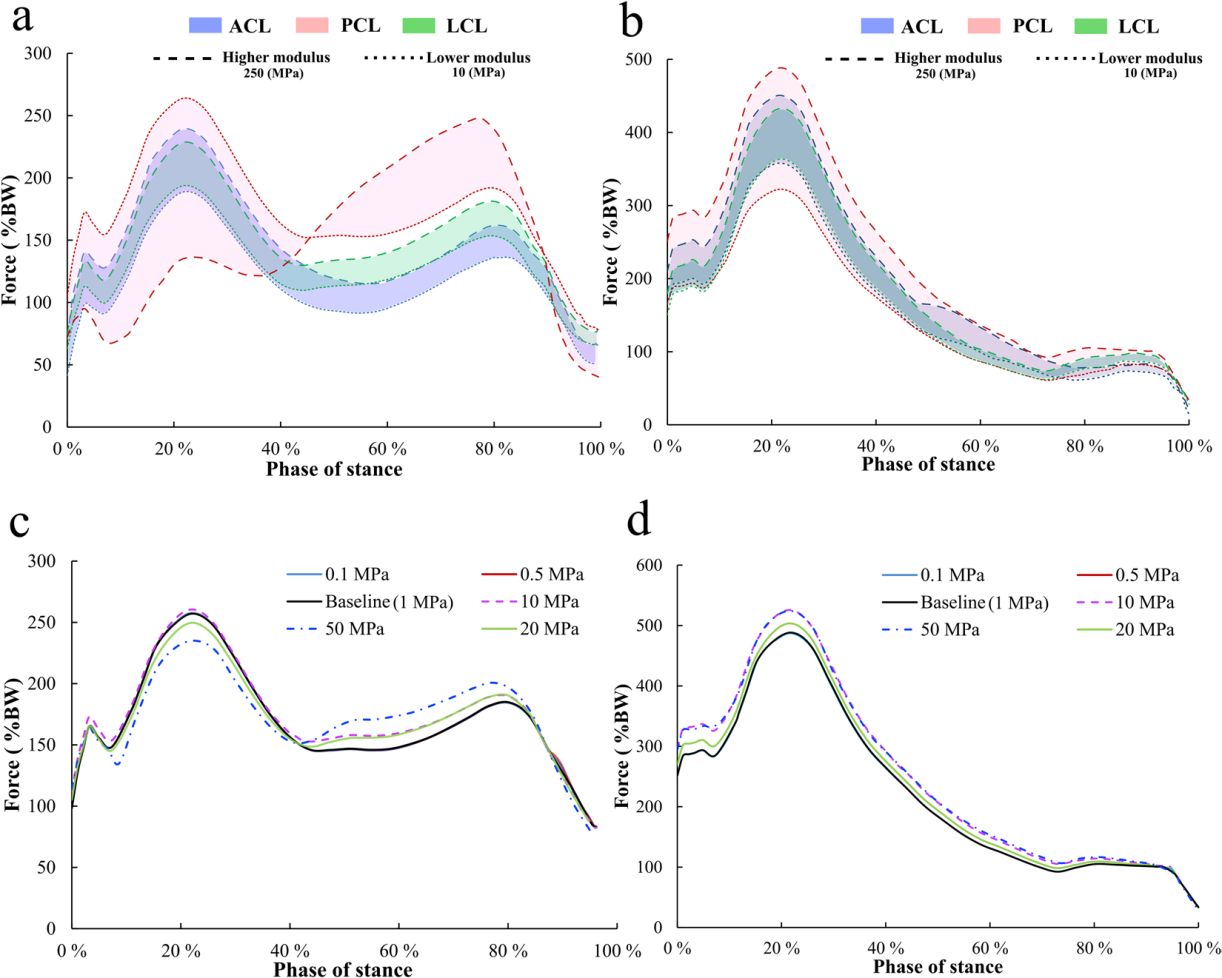
Tibiofemoral (**a**) and patellofemoral joint reaction forces (**b**) in the FRPHE model after parametric variation of the fibrillar network modulus in ligaments (Table 2), while the other model parameters were kept unchanged (Table 1). Tibiofemoral (**c**) and patellofemoral joint reaction forces (**d**) in the FRPHE model after parametric variation of the nonfibrillar modulus in ligaments from 0.1 to 50 MPa, while the other material parameters were kept unchanged (Table 1).

Parametric analysis within the FRPHE model by varying the fibril network modulus in every ligament (Table 2) showed a considerable influence of PCL, ACL and LCL on tibial and patellar forces (Fig. 8a and b); minimal impact was observed by the rest of the ligaments (data not shown). Variation of the PCL fibril network modulus showed the largest changes; increasing the modulus reduced the first peak force at the tibiofemoral contact and increased the second peak but decreasing the modulus value caused an opposite behavior. On the other hand, the fibril network modulus of the ACL and LCL modulated joint forces similarly during the entire stance phase; increasing the fibril network modulus increased tibiofemoral joint forces consistently (Fig. 8a). Modifications in the PCL, ACL, and LCL fibril network properties demonstrated similar effect in the force on patellar cartilage; with the chosen range of the fibril network modulus values, PCL controlled mostly this force as well (Fig. 8b).

In contrast, variation of the nonfibrillar matrix modulus in all ligaments exhibited only a small influence on the tibiofemoral and patellofemoral reaction forces; increasing substantially the nonfibrillar matrix modulus reduced the first peak force at the tibiofemoral contact but augmented the forces after ~40% of the phase stance (Fig. 8c). In addition, variations in the initial permeability *k*_0_ and material constant *M*, as well as the two boundary conditions on the external surface of ligaments (sealed and free draining) in the FRPHE model, did not change the results on the tibiofemoral contact (the maximum difference in joint reaction forces was 2%).

## Discussion

In the present study, computational analysis showed that different geometrical representations (springs versus solid) and constitutive formulations of ligaments, particularly the compression-tension behavior, affect the human knee motion and tibial cartilage responses. This was mainly explained by the fibril-reinforcement attribute causing large tensile stresses and almost negligible compressive stresses. On the other hand and with a proper choice of material parameters, during the first ~50% of the stance all constitutive models could produce similar results. Further parametric analysis also demonstrated that the effect of the fibril network modulus of ligaments on the joint forces was predominant, and its variation, particularly in the PCL, modulated joint forces substantially. The nonfibrillar matrix modulus of ligaments had only a small influence on the forces, while fluid flow had virtually no influence on the joint forces and cartilage responses.

The original model with cartilage and menisci meshed, ligaments modeled as springs, and knee motion implemented was developed earlier and compared to experiments^22,44^. In the present study, we generated new ligament and tendon geometries for this model and implemented several different constitutive models for ligaments. For these reasons, we only briefly mention here how the current model results compare with experiments and computational studies. The magnitude of the joint reaction forces (2–3 BW) obtained from all the generated numerical knee models agree with recent experimental data^45–47^ and numerical studies^48,49^. Knee motion, contact pressures, and cartilage stresses and strains were also consistent with several earlier experimental and computational studies^29,50–52^. Varus-valgus and internal-external rotations matched well with the measured values of the same subject. Find more details about the validation of this model from the supplementary material.

Although the contribution of the compressive properties of ligaments may occur to some extent *in vivo* where ligaments are exposed to multiaxial states of stresses, bending, and transverse compressive loads during locomotion^26–28^, it is evident that this contribution is much smaller than that in tension^12,14,29^. The FRPHE model was able to capture this compression-tension characteristic, thereby giving slightly different knee joint forces and cartilage responses during loading as compared to the other solid models without this feature. The effect of the lack of the compressive properties of ligaments with the fibril-reinforced model was particularly observed at the end of stance, where particularly the PCL experienced also bending and subsequent local compressive loads and then the force obviously was slightly higher in the models without this compression-tension difference in the ligaments. Yet, local tensile stresses also in these simpler solid models were substantially higher than local compressive stresses because ligaments obviously are primarily in tension during gait.

In terms of joint reaction forces, the simplest model with spring elements representing ligaments was actually the closest match with the FRPHE model. The reason for this is that this model has no compressive resistance to load. The difference between these models came from the ligament geometry and nonfibrillar matrix contribution in the FRPHE model. Particularly, in ligaments as 3D continuum, cruciate ligaments had varying degrees of twisting during loading and collateral ligaments bent dissimilarly and inhomogeneously throughout the knee motion but especially from 50% to 90% of the stance. This complex interplay caused changes in length and fibril orientation of ligaments, causing combined loading states in the ligaments e.g. shear and bending, and showing an intricate interaction between the fibril network and nonfibrillar matrix. Moreover, the FRPHE model could produce the same response with the rest of models during certain time points of the stance. These findings indicate that simpler formulations of ligaments could be used to solve particular biomechanical problems, without the need to develop an elaborated constitutive representation for ligaments.

Tibial lateral and anterior translations and external rotation were the most sensitive kinematic parameters under varying ligament constitutive models which also contributed to the different joint reaction forces particularly at about 50 to 100% of the stance. Complex mechanical interactions in and between solid cruciate ligaments provided a reduced elongation and therefore smaller translation. In contrast, spring ligaments possessed more space to elongate, consequently this simplified model experienced higher translations. Additionally, FRPHE ligaments were able to bend and were supported partly by tensile forces at certain edges and virtually no compressive loads, while spring elements, which are constantly loaded along their axis, have a reduced capacity to twist during the knee motion. Interestingly, fibers in ligaments as 3D continuum contributed to reduce the elongation of ligaments, and accordingly the FRPHE model exhibited slightly different medial-lateral translation and smaller external-internal rotation regarding the other three-dimensional models for ligaments.

Due to slightly different knee motion in the FRPHE model compared to the other models, the tibiofemoral contact area was relocated in the medial tibial plateau and the contact area increased slightly. Therefore, the average contact and fluid pressures, and maximum principal strain in cartilage were smaller in the fibril-reinforced model, while elevated average fibril strains and tensile stresses were generated in the cartilage contact area. On the other hand, and primarily due to different medial-lateral translation, stresses, strains, and contact and fluid pressures in the lateral joint compartment were the highest in the FRPHE model.

It is known that the mechanical properties of ACL, PCL, and LCL have a notable effect on the biomechanical response of the knee joint^18,20,52^. Our parametric study with the FRPHE model showed also that tibiofemoral and patellofemoral joint forces were strongly influenced by the collagen network modulus of ACL, PCL, and LCL. This result is consistent with numerical studies which have examined the integrated interplay of knee ligaments^29,52–54^. Interestingly, while increasing the fibril network modulus of both the ACL and LCL increased joint reaction forces consistently throughout the gait, by increasing the fibril network modulus of the PCL first reduced the joint reaction forces (till ~50% of stance) and then increased the forces (from ~50% till 90% of stance) above the other models. On the other hand, reduction in the PCL fibril network modulus caused increased joint reactions forces through the stance. This implies an increase of anterior tibial translation and shift of the tibiofemoral contact area toward posterior direction, increasing the stress concentration in specific areas of the tibial cartilage. In addition, the ACL influence is consistent with our previous study^22^ and the remarkable PCL impact is congruent with recent studies^55–58^. In addition, this particular PCL influence is consistent with experimental studies that have reported a significant increase in joint contact forces and pressure concentrations on the medial compartment in a posterior cruciate deficient knee^59,60^. This could be the mechanism that causes joint degeneration after PCL deficiency^58^.

The parametric results within the FRPHE model suggest that fluid flow of ligaments (permeability and fluid flow boundary condition) has a negligible role for the cartilage response during the knee joint motion primarily due to tensile forces generated on ligaments. This result is consistent with several earlier studies on soft tissues, suggesting that the transient response of these tissues in tension is controlled by intrinsic properties of the solid matrix^36,61,62^, not by fluid flow which controls the transient response in compression^63,64^. Consistently, the effect of the nonfibrillar matrix modulus (range 0.1–50 MPa) on knee joint forces was small. This is also consistent with several other studies on fibril-reinforced poroelastic soft tissues^36,65–68^, and is a result of the high ratio between the fibril network and nonfibrillar matrix moduli.

Since the density ratio between the primary, organized fibrils and secondary, randomly organized fibrils (*C*) in the FRPHE models is not known for ligaments, in ACL and PCL it was assumed to be the same than that given for cartilage. However, lower *C* values had to be given for MCL and LCL since high values in these ligaments started to control the end part of the stance differently than with any other model. Since the collagen network stress (Eq. 9) is controlled by both the fibril network modulus and *C*, alternative combinations of these values could give the same result. Therefore, the modulus values in the fibril-reinforced model should not be directly related to the elastic tensile modulus values obtained from experiments.

Some limitations exist in this study regarding the model generation, input and assumptions. First, tissue geometries and gait input data were based on a single healthy male subject, as obtained from an earlier study^22^, except ligaments and tendons which were segmented separately for the purposes of this investigation. We also modeled only walking because it is the most typical type of movement. However, the fundamental behavior of ligaments should not change by modeling another subject and this methodology can be easily extended for other daily motor tasks (e.g. stair climbing, sit-to-stand, squatting). Second, the ligament pre-strains were taken from an earlier numerical study^69^ although PCL might be lax and it may not be active at full extension^17,70^. Also ligament collagenous part was simplified (bilinear) and not fully nonlinear with the toe region as has been presented in the literature^71^ because ligaments were assumed to function primarily at the linear region in the knee^8,30^. However, the ligament pre-strains and bilinearities were assumed to be the same for all the models analyzed in this study and the conclusion about the effect of different material representations should not change by different pre-strains and ligament properties. Though, if the PCL strain would be much smaller during loading, then likely the contribution of the fibril network modulus of the PCL would not be that drastic. Nonetheless, with these assumptions the maximum tibial reaction forces obtained (2–3 BW) concurred well with selected previous studies^46,48^.

The run time for the spring model was shorter than that for the FRPHE model but longer than that for the hyperelastic model. Also, the pre-processing steps for the spring model were faster than those for the hyperelastic and FRPHE models due to segmentation. On the other hand, the development and implementation of the fibril-reinforced properties of ligaments is demanding and time-consuming. These differences might be important in medical applications. However, as shown by the results, simpler models do not necessarily produce the same results with more complex fibril-reinforced models throughout the stance phase. Hence, simple representations for tissues could be used if they produce results close to the more realistic fibril-reinforced materials^72^ in certain steps of the phase stance. Moreover, automatic segmentation and meshing techniques would also be desirable to speed up the creation of the knee models^73,74^. The models with higher complexity might be better for investigation of local changes in the tissues such as rupture and biophysical adaptations of ligaments, tendons, cartilage and menisci.

In conclusion, the results of the current study suggest that the compression-tension relationship in ligaments due to the fibril-reinforcement contributes substantially to the knee joint motion and forces as well as cartilage responses during stance, while the roles of the nonfibrillar matrix and fluid are small or negligible. On the other hand, at certain phases of stance and with a proper choice of material parameters, knee models with simpler material models of ligaments (either springs or other solid constitutive models) are suggested to be able to reproduce similar results. These findings and suggestions are relevant to consider in biomechanical models to explore treatments (surgical or conservative) associated with knee ligament injuries.

## Electronic supplementary material

Supplementary material
