# Supplementary material for: The effect of constitutive representations and structural constituents of ligaments on knee joint mechanics
Source: Sci Rep. 2018 Feb 2;8:2323. doi: 10.1038/s41598-018-20739-w (PMC5797142; doi:10.1038/s41598-018-20739-w)
Supplement: Supplementary file 1 — Supplementary material [file 41598_2018_20739_MOESM1_ESM.pdf]

Supplementary material

The effect of constitutive representations and structural constituents of ligaments on knee joint mechanics

Gustavo A. Orozco[1], Petri Tanska[1], Mika E. Mononen[1], Kimmo Halonen[2] and Rami K. Korhonen[1]

[1]*Department of Applied Physics, University of Eastern Finland, Kuopio, Finland*
[2]*Department of Health Science and Technology, Aalborg University, Aalborg, Denmark*

Methods

Finite element model

The ligaments in the knee models were assumed to be in pre-tension at their segmented length in MRI data; hence according to an earlier study, we imposed a pre-strain of 5% on ACL and PCL and 4% on LCL and MCL[1–3]. We implemented the pre-strains using the predefined field stress option in Abaqus (the equivalent stress tensor to the pre-strain was calculated and applied in each ligament). In addition, the depth-dependent arcade-like collagen fibril architecture with split-line patterns was implemented in the cartilages while the fibrils were oriented circumferentially in the menisci. Moreover, we applied split-lines in the patellar cartilage along with the superior-inferior direction[4]. See previous studies for more details about the materials in the model[5–9]. For the full list of material parameters of cartilages and menisci, see Table 1 in[10].

Implementation for different constitutive models for ligaments

Five knee joint models were constructed with different constitutive models for the ligaments: 1) spring, 2) linear elastic, 3) hyperelastic, 4) porohyperelastic and 5) fibril-reinforced porohyperelastic (FRPHE) material, which we briefly describe here. In all cases, stress-strain behavior of the ligaments was defined such that the ligaments produced force in

tension (strain > 0) but not in compression (strain < 0) during the gait. For the first two cases, Hooke´s law represents the relation between stresses (or forces) and strains (or elongations)

$$\boldsymbol{\sigma}_{\text{tot}} = \mathbf{C}\,\boldsymbol{\varepsilon}\,, \tag{1}$$

where $\boldsymbol{\sigma}_{\mathbf{tot}}$ is the Cauchy stress tensor, $\boldsymbol{\varepsilon}$ is the infinitesimal strain tensor, and $\mathbf{C}$ is the fourth-order stiffness matrix, which is defined by Young's modulus (E) and Poisson's ratio (ν). For the spring model, the stiffness is defined by a spring constant k. The third model was defined using a neo-Hookean material, in which the stresses are given by

$$\boldsymbol{\sigma}_{\text{tot}} = K_{\text{m}}\frac{\ln(J)}{J}\mathbf{I} + \frac{G_{\text{m}}}{J}\left(\mathbf{F}\cdot\mathbf{F}^{\text{T}} - J^{\frac{2}{3}}\,\mathbf{I}\right), \tag{2}$$

where K_{m} is the bulk modulus, G_{m} is the shear modulus, J is the determinant of the deformation gradient tensor $\mathbf{F}$ and $\mathbf{I}$ is the unit tensor. The bulk (K_{m}) and shear (G_{m}) modulus are defined as

$$K_{\text{m}} = \frac{E}{3(1-2\nu)}\,, \tag{3}$$

$$G_{\text{m}} = \frac{E}{2(1+\nu)}\,. \tag{4}$$

For the fourth model, the ligaments were described as a biphasic tissue in which the porous solid matrix is fully saturated with water. The total stress in the tissue is then given by

$$\boldsymbol{\sigma}_{\text{tot}} = \boldsymbol{\sigma}_{\text{s}} + \boldsymbol{\sigma}_{\text{fl}} = \boldsymbol{\sigma}_{\text{eff}} - p\mathbf{I}\,, \tag{5}$$

where $\boldsymbol{\sigma}_{\text{tot}}$ is the total stress tensor, $\boldsymbol{\sigma}_{\text{s}}$ is the stress in the solid matrix, $\boldsymbol{\sigma}_{\text{fl}}$ is the stress in the fluid matrix, p is the hydrostatic pressure and $\boldsymbol{\sigma}_{\text{eff}}$ is the effective solid stress. In this model, $\boldsymbol{\sigma}_{\text{eff}}$ was described by equation (2). Additionally, the permeability k was assumed to be strain-dependent according to[11] and is as follows:

$$k = k_0\left[\frac{\phi_0\phi_{\text{f}}}{(1-\phi_0)\phi_{\text{s}}}\right]^2 \exp\left(\frac{M(J^2-1)}{2}\right), \tag{6}$$

where k_0 is the initial permeability, M is a positive constant, ϕ_0 is the initial volume fraction of the solid phase, ϕ_{f} is the current volume fraction of the fluid phase and ϕ_{s} is the current

volume fraction of the solid phase. Finally, the FRPHE model considers that the solid matrix is divided into a non-fibrillar part, describing primarily the proteoglycan matrix, and a fibrillar elastic network, representing the collagen fibers. The total stress in the ligament tissue is then given by

$$\boldsymbol{\sigma}_{\mathrm{tot}} = \boldsymbol{\sigma}_{\mathrm{s}} - p\mathbf{I} = \boldsymbol{\sigma}_{\mathrm{f}} + \boldsymbol{\sigma}_{\mathrm{nf}} - p\mathbf{I}\,, \tag{7}$$

where $\boldsymbol{\sigma}_{\mathrm{f}}$ and $\boldsymbol{\sigma}_{\mathrm{nf}}$ are the stresses in the collagen fibers and the non-fibrillar matrix, respectively. The non-fibrillar component of the ligament is defined using a neo-Hookean material with biphasic properties as was described in equation (5). The fibril stress σ_{f} is given by

$$\sigma_{\mathrm{f}} = \begin{cases} E_{\mathrm{f}}\varepsilon_{\mathrm{f}}\,, & \varepsilon_{\mathrm{f}} > 0 \\ 0\,, & \varepsilon_{\mathrm{f}} \leq 0 \end{cases}\,, \tag{8}$$

where E_{f} is the fibril network modulus and ε_{f} is the fibril strain. Note that these fibrils resist only tensile forces. The fibril network stress arises from the sum of primary and secondary collagen fibril stresses, which is calculated separately for each integration point in each element[9]. Stresses for these fibrils in tension were

$$\begin{cases} \sigma_{\mathrm{f,p}} = \rho_z C \sigma_{\mathrm{f}} \\ \sigma_{\mathrm{f,s}} = \rho_z \sigma_{\mathrm{f}} \end{cases}\,, \tag{9}$$

where $\sigma_{\mathrm{f,p}}$ and $\sigma_{\mathrm{f,s}}$ are the fibril stresses for primary and secondary fibrils, respectively, C is the density ratio between primary and secondary fibrils and ρ_z is the relative collagen density. We implemented primary fiber family along with the longitudinal direction of ligaments using the method described in[6], whereas secondary fibrils possessed a random orientation in order to replicate collagen crosslinks[12,13]. Orientations were implemented by using a self-made function in Matlab (The MathWorks, Natick, MA, USA).

Results

Validation

In accordance with subject-specific and other experimental data[14,15], external-internal rotations showed similar tendencies (Fig. 1a). The extension-flexion rotations matched closely to the literature values. Note that identical behavior was obtained among the models because extension-flexion rotation was used as an input parameter (Fig. 1b). The models yielded similar trend in the valgus-varus rotations compared to the earlier experimental values and subject-specific data. Dissimilarities in the values might be related with anatomical variations (Fig. 1c). On the other hand, tibiofemoral joint reaction forces in the models were very consistent with the literature[16–18] with some variations in the forces (Fig. 1d). On the other hand, the values of the maximum joint contact pressures of the medial tibial cartilage were in close agreement with the experimental data and a numerical study reported in the literature[19,20] (Fig. 2).

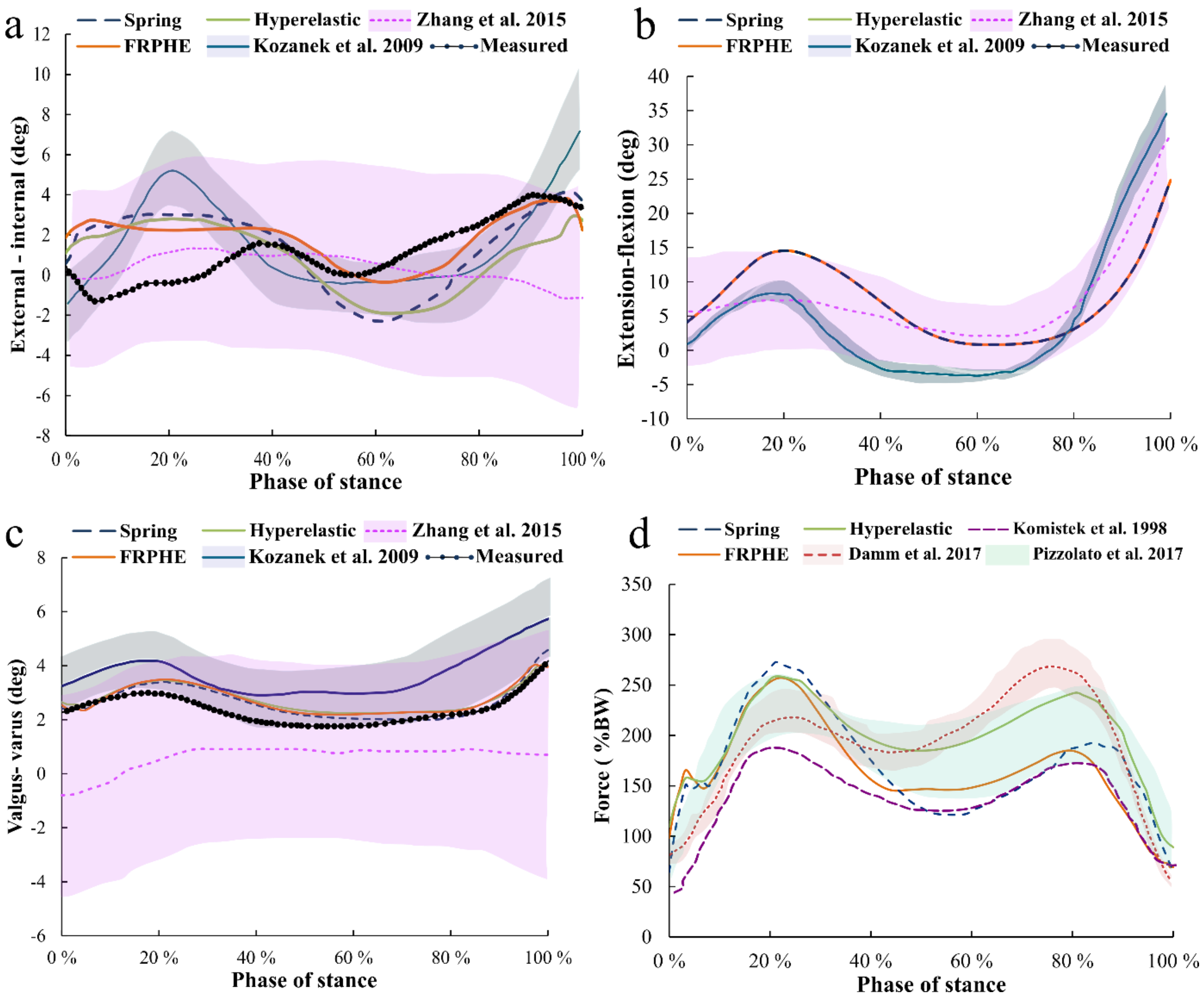

Fig. 1. Comparisons of tibial rotations and tibiofemoral reaction forces during the stance phase with respect to literature reports and subject-specific data (measured: motion analysis of the modeled subject combined with musculoskeletal modeling[2]). (a) External-internal rotation. (b)

Extension-flexion rotation. Note that extension-flexion rotations are identical in all knee models because they were used as an input parameter. (c) Valgus-varus rotation. The blue shaded areas denote the maximal and minimal rotations[14]. The dashed pink lines represent the subjects' average motion and the shaded pink areas represent the standard deviation[15]. (d) Tibiofemoral joint reaction forces in percent of bodyweight (%BW). The red shaded areas represent the range between 25th and 75th percentiles of force patterns reported[16]. The green shaded areas represent the minimum-maximum range of the measured forces[18].

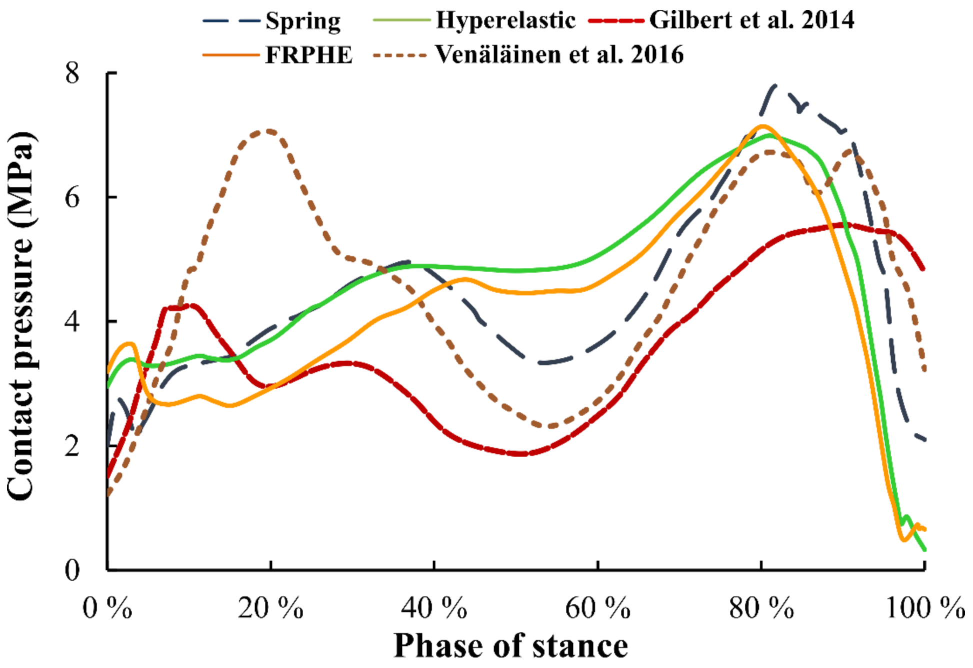

Fig. 2. Comparisons of maximum contact pressures of the medial tibial cartilage with respect to numerical[20] and experimental[19] contact pressure values from the literature.

Discussion

Since the material properties of ligaments and subject-specific contact forces cannot be measured *in vivo*, literature values were used to validate the numerical models. These comparisons showed a close agreement with the experimental data. The current models with the material properties from literature matched also quite well with the rotations measured for this same subject.

In the 3D continuum models for ligaments the contact stresses between solid ligaments (i.e. ACL and PCL) were quite low. Although an array of spring elements in the simplified model of ligaments was implemented, this approach does not take into account the contact interactions among ligaments attributable to 1D element limitations. Therefore, this different ligament contact might slightly contribute to the differences between the spring and solid models (see also the 5th paragraph of the discussion from the manuscript). However, other aspects such as pre-strains, ligament insertion sites and bilinearities were assumed to be the same for all the models.

References

1. Gantoi, F. M., Brown, M. A. & Shabana, A. A. Finite Element Modeling of the Contact Geometry and Deformation in Biomechanics Applications1. *J. Comput. Nonlinear Dyn.* **8,** 041013–041013 (2013).
2. Halonen, K. S. *et al.* Importance of Patella, Quadriceps Forces, and Depthwise Cartilage Structure on Knee Joint Motion and Cartilage Response During Gait. *J. Biomech. Eng.* **138,** (2016).
3. Halonen, K. S. *et al.* Optimal graft stiffness and pre-strain restore normal joint motion and cartilage responses in ACL reconstructed knee. *J. Biomech.* **49,** 2566–2576 (2016).
4. Bae, W. C. *et al.* Wear-lines and split-lines of human patellar cartilage: relation to tensile biomechanical properties. *Osteoarthritis Cartilage* **16,** 841–845 (2008).
5. Halonen, K. S., Mononen, M. E., Jurvelin, J. S., Töyräs, J. & Korhonen, R. K. Importance of depth-wise distribution of collagen and proteoglycans in articular cartilage—A 3D finite element study of stresses and strains in human knee joint. *J. Biomech.* **46,** 1184–1192 (2013).

6. Mononen, M. E. *et al.* Effect of superficial collagen patterns and fibrillation of femoral articular cartilage on knee joint mechanics-a 3D finite element analysis. *J. Biomech.* **45,** 579–587 (2012).
7. Wilson, W., van Donkelaar, C. C. & Huyghe, J. M. A comparison between mechano-electrochemical and biphasic swelling theories for soft hydrated tissues. *J. Biomech. Eng.* **127,** 158–165 (2005).
8. Julkunen, P., Kiviranta, P., Wilson, W., Jurvelin, J. S. & Korhonen, R. K. Characterization of articular cartilage by combining microscopic analysis with a fibril-reinforced finite-element model. *J. Biomech.* **40,** 1862–1870 (2007).
9. Wilson, W., van Donkelaar, C. C., van Rietbergen, B., Ito, K. & Huiskes, R. Stresses in the local collagen network of articular cartilage: a poroviscoelastic fibril-reinforced finite element study. *J. Biomech.* **37,** 357–366 (2004).
10. Halonen, K. S. *et al.* Deformation of articular cartilage during static loading of a knee joint--experimental and finite element analysis. *J. Biomech.* **47,** 2467–2474 (2014).
11. Weiss, J. A. & Maakestad, B. J. Permeability of human medial collateral ligament in compression transverse to the collagen fiber direction. *J. Biomech.* **39,** 276–283 (2006).
12. Eleswarapu, S. V., Responte, D. J. & Athanasiou, K. A. Tensile Properties, Collagen Content, and Crosslinks in Connective Tissues of the Immature Knee Joint. *PLOS ONE* **6,** e26178 (2011).
13. Hanada, M., Takahashi, M., Suzuki, D., Abe, M. & Matsuyama, Y. A biochemical study of the distribution of collagen and its crosslinks in knee ligaments and the patellar tendon. *Connect. Tissue Res.* **55,** 378–383 (2014).
14. Kozanek, M. *et al.* Tibiofemoral kinematics and condylar motion during the stance phase of gait. *J. Biomech.* **42,** 1877–1884 (2009).
15. Zhang, Y. *et al.* Motion analysis of Chinese normal knees during gait based on a novel portable system. *Gait Posture* **41,** 763–768 (2015).

16. Damm, P., Kutzner, I., Bergmann, G., Rohlmann, A. & Schmidt, H. Comparison of in vivo measured loads in knee, hip and spinal implants during level walking. *J. Biomech.* **51,** 128–132 (2017).

17. Komistek, R. D., Stiehl, J. B., Dennis, D. A., Paxson, R. D. & Soutas-Little, R. W. Mathematical model of the lower extremity joint reaction forces using Kane's method of dynamics. *J. Biomech.* **31,** 185–189 (1997).

18. Pizzolato, C. *et al.* Biofeedback for Gait Retraining Based on Real-Time Estimation of Tibiofemoral Joint Contact Forces. *IEEE Trans. Neural Syst. Rehabil. Eng.* **25,** 1612–1621 (2017).

19. Gilbert, S. *et al.* Dynamic contact mechanics on the tibial plateau of the human knee during activities of daily living. *J. Biomech.* **47,** 2006–2012 (2014).

20. Venäläinen, M. S. *et al.* Quantitative Evaluation of the Mechanical Risks Caused by Focal Cartilage Defects in the Knee. *Sci. Rep.* **6,** 37538 (2016).
